# Association of Kidney Function With Development of Alzheimer Disease and Other Dementias and Dementia-Related Blood Biomarkers

**DOI:** 10.1001/jamanetworkopen.2022.52387

**Published:** 2023-01-24

**Authors:** Hannah Stocker, Léon Beyer, Kira Trares, Laura Perna, Dan Rujescu, Bernd Holleczek, Konrad Beyreuther, Klaus Gerwert, Ben Schöttker, Hermann Brenner

**Affiliations:** 1 Network Aging Research, Heidelberg University, Heidelberg, Germany; 2 Division of Clinical Epidemiology and Aging Research, German Cancer Research Center, Heidelberg, Germany; 3 Population Health Sciences, German Center for Neurodegenerative Diseases, Bonn, Germany; 4 Center for Protein Diagnostics (ProDi), Ruhr-University Bochum, Bochum, Germany; 5 Faculty of Biology and Biotechnology, Department of Biophysics, Ruhr-University Bochum, Bochum, Germany; 6 Department of Translational Research in Psychiatry, Max Planck Institute of Psychiatry, Munich, Germany; 7 Department of Psychiatry, University of Vienna, Vienna, Austria; 8 Saarland Cancer Registry, Saarbrücken, Germany

## Abstract

**Importance:**

Previous research has suggested an association of kidney function with risk of Alzheimer disease (AD) or other dementias and dementia-related blood biomarkers, but a distinct association remains unclear.

**Objective:**

To evaluate the association of kidney function with risk of diagnosis of incident AD or dementia within 17 years and with the blood biomarkers neurofilament light (NfL), phosphorylated tau181 (p-tau181), and glial fibrillary acidic protein (GFAP).

**Design, Setting, and Participants:**

In this prospective, population-based cohort study and nested case-control study, 9940 participants in Germany were enrolled between 2000 and 2002 by their general practitioners and followed up for up to 17 years. Participants were included if information on dementia status and creatinine/cystatin C measurements were available. A subsample of participants additionally had measurements of NfL, p-tau181, and GFAP obtained from blood samples. Statistical analysis was performed from January 3 to November 25, 2022.

**Exposures:**

Impaired kidney function, based on estimated glomerular filtration rate less than 60 mL/min/1.73 m^2^ according to the 2021 Chronic Kidney Disease Epidemiology Collaboration creatinine–cystatin C equation.

**Main Outcomes and Measures:**

All-cause dementia, AD, and vascular dementia diagnosis, as well as log-transformed levels of NfL, p-tau181, and GFAP in blood.

**Results:**

Of 6256 participants (3402 women [54.4%]; mean [SD] age at baseline, 61.7 [6.6] years), 510 received an all-cause dementia diagnosis within 17 years of baseline. The dementia-related blood biomarker nested case-control sample included 766 participants. After adjusting for age and sex, impaired kidney function at baseline was not associated with a higher risk of all-cause dementia (hazard ratio [HR], 0.95; 95% CI, 0.69-1.29), AD (HR, 0.94; 95% CI, 0.55-1.63), or vascular dementia diagnosis (HR, 1.06; 95% CI, 0.65-1.70) within 17 years. In the cross-sectional analysis, after adjusting for age and sex, impaired kidney function was significantly associated with NfL and p-tau181 levels in blood (NfL: β = 0.47 and *P* < .001; p-tau181: β = 0.21 and *P* = .003). After adjusting for age and sex, significant associations with GFAP levels were evident only among men (men: β = 0.31 and *P* = .006; women: β = −0.12 and *P* = .11).

**Conclusions and Relevance:**

In this population-based study of community-dwelling adults, reduced kidney function was associated with increased levels of dementia-related blood biomarkers but not increased dementia risk. Kidney function might influence the accuracy of dementia-related blood biomarkers and should be considered in clinical translation.

## Key Points

**Question:**

Is kidney function associated with Alzheimer disease (AD) or other dementias or with dementia-related blood biomarkers (neurofilament light [NfL], phosphorylated tau181 [p-tau181], and glial fibrillary acidic protein [GFAP])?

**Findings:**

In this population-based cohort study of 6256 individuals, kidney function was not associated with risk of dementia diagnosis, including AD and vascular dementia subtypes, within 17 years, but was associated with blood biomarker levels of NfL and p-tau181. A sex-specific association between kidney function and GFAP levels in blood was apparent, with significant associations evident only among men.

**Meaning:**

This study suggests that reduced kidney function may be associated with increased levels of dementia-related blood biomarkers but not necessarily with increased AD or dementia risk.

## Teaser

This cohort study evaluates the association of kidney function with risk of diagnosis of incident Alzheimer disease or dementia and with the blood biomarkers neurofilament light, phosphorylated tau, and glial fibrillary acidic protein.

## Introduction

Alzheimer disease (AD), the most common form of dementia, has a long preclinical phase, and the cause and progression of the disease are multifactorial.^1,2^ Increased understanding of the disease is critical to effective treatment, prevention, and use of AD biomarkers in the clinical setting. Kidney function has been previously associated with risk of dementia, including AD and vascular dementia.^3,4,5^ The literature regarding this association, however, has been inconsistent, with several studies showing no association between kidney function and dementia risk or cognitive decline.^6,7,8^

Although very limited research exists regarding factors associated with dementia-related blood biomarkers, kidney function has also been shown in several studies to be associated with biomarker levels and has been named a research priority in recent recommendations by the Alzheimer’s Association on the appropriate use of blood biomarkers.^9^ Reduced kidney function has been associated with increased levels of neurofilament light (NfL),^10,11,12^ phosphorylated tau (p-tau),^12,13^ total tau,^10^ and amyloid-β in blood.^10^

Dementia-related blood biomarkers have shown excellent capability in AD pathology and diagnosis discrimination.^14^ To assess the future of these biomarkers in the clinical setting, the identification of influential factors such as kidney disease, which has an estimated global prevalence of 9.1% or roughly 700 million cases,^15^ is crucial to the establishment of clinically useful ranges and interpretation of blood biomarker levels.

Considering the kidneys are responsible for blood filtration and the mechanism linking kidney function to AD is unclear, it is important to explore more extensively whether kidney function plays an influential role in AD pathology or is merely a factor associated with the accuracy of blood AD biomarkers.

The aim of this study was to explore the association of kidney function with risk of incident AD or dementia diagnosis within 17 years and with the dementia-related blood biomarkers NfL, p-tau181, and glial fibrillary acidic protein (GFAP) in a prospective community-based cohort study. Secondarily, sex- and age-stratified analyses were conducted.

## Methods

### Study Participants and Data Collection

The analyses are based on a nested case-control study within the ESTHER (Epidemiologische Studie zu Chancen der Verhütung, Früherkennung und optimierten THerapie chronischer ERkrankungen in der älteren Bevölkerung) study, a population-based prospective cohort study of community-dwelling older adults in Germany. In brief, the ESTHER study consists of 9940 participants recruited by general practitioners in the context of a general health screening examination in a statewide study in Saarland, a small state (approximately 1 million inhabitants) located in southwest Germany, from 2000 to 2002.^16,17^ The inclusion criteria of the ESTHER study were age 50 to 75 years and proficiency of the German language to provide written informed consent. The ESTHER study was approved by the ethics committee of the medical faculty at Heidelberg University and the Physicians’ Board of Saarland. The report of this study followed the Strengthening the Reporting of Observational Studies in Epidemiology (STROBE) reporting guideline.

Data on dementia diagnoses were collected from participants’ general practitioners, who were asked to fill out questionnaires and provide all available medical records regarding dementia diagnoses (or lack of dementia diagnoses) throughout follow-up.^17,18^ There was no specific question regarding race and ethnicity in the baseline questionnaire. However, European descent was determined through genetic analysis. More details regarding the ESTHER study and the dementia diagnoses can be found in eAppendix 1 in Supplement 1. The sample for this study included ESTHER participants with available dementia information and baseline serum creatinine and serum cystatin C measurements (N = 6256) (eFigure 1 in Supplement 1). The analyses with the AD-related blood biomarkers (p-tau181, GFAP, and NfL) were conducted within a previously defined nested case-control study (n = 768) in the ESTHER study that included all participants with a diagnosis of AD between baseline and the 17-year follow-up, several patients with incident vascular dementia and mixed dementia diagnoses for comparison, and randomly selected controls.^19^ In this study, participants without baseline serum creatinine and serum cystatin C measurements were excluded (n = 2) (eFigure 1 in Supplement 1).

### Data Ascertainment at Baseline and Laboratory Measurements

Demographic, medical, and lifestyle information was ascertained at baseline through self-administered questionnaires and/or physician reports. In addition, serum creatinine, cystatin C, and additional laboratory test measurements were obtained from blood and urine samples. The p-tau181, GFAP, and NfL measurements were completed using Simoa (singular molecular array) technology as previously described.^19^ Details regarding data ascertainment and laboratory test measurements can be found in eAppendix 2 in Supplement 1.

Kidney function was assessed through the estimated glomerular filtration rate (eGFR). The eGFR was calculated using the 2021 Chronic Kidney Disease Epidemiology Collaboration creatinine–cystatin C (eGFRcr-cys) equation^20^: eGFRcr-cys = μ × min [(Scr/*k*), 1]*^a^*^1^ × max [(Scr/*k*), 1]*^a^*^2^ × min [(Scys/0.8), 1]*^b^*^1^ × max [(Scys/0.8), 1]*^b^*^2^ × *c*^age^ × *d* (if female).

Kidney function was defined categorically as normal (≥90 mL/min/1.73 m^2^), mildly decreased (60-89 mL/min/1.73 m^2^), and impaired (<60 mL/min/1.73 m^2^). Too few participants had an eGFR less than 30 mL/min/1.73 m^2^ to add a category for kidney failure.

### Statistical Analysis

Statistical analysis was performed from January 3 to November 25, 2022. Baseline characteristics of participants using summary statistics were calculated for the sample included in the study. Cumulative incidence curves stratified by kidney function were calculated for all-cause dementia, AD, and vascular dementia diagnoses within 17 years. Multiple imputation (n = 20) for data missing at random was conducted using the Markov chain Monte Carlo method with all included variables. The imputed variables and the percentage of missing data are listed in eTable 1 in Supplement 1.

The longitudinal analysis investigated the association between kidney function, as defined by the eGFRcr-cys, and dementia diagnosis (AD, vascular dementia, or all-cause dementia). Cox proportional hazards regression using the imputed data sets was conducted to calculate hazard ratios (HRs) and 95% CIs, with incidence of all-cause dementia including subtypes as the dependent variables. The end of the observation included the date of dementia diagnosis, date of death, or date of the 17-year follow-up (date of response from the general practitioner regarding dementia status). The proportionality assumption for the Cox proportional hazards regression models was assessed using the methods outlined by Lin et al.^21^ Multicollinearity and interaction were assessed using variance inflation factors and interaction tests to determine the appropriate covariates for each model. Multivariable analysis with various levels of adjustment was conducted to reflect overall prognostic ability and independent association with the prognosis of dementia risk. Model 0 represents the crude associations, model 1 is adjusted for age and sex, and model 2 included all of the following covariates: age, sex, educational level, physical activity level, lifetime history of depression, stroke, any cancer, myocardial infarction, hypertension, diabetes, congestive heart failure, body mass index (BMI; calculated as weight in kilograms divided by height in meters squared), smoking status, alcohol use, cholesterol level, C-reactive protein level, 8-iso-prostaglandin F_2α_ level, various medication use (nonsteroidal anti-inflammatory drugs, angiotensin-converting enzyme inhibitors, diuretics, calcium channel blockers, β-blockers, and statins), and 25(OH)D level. Sensitivity analyses were conducted including models that were only age adjusted or only sex adjusted and a more restricted full model that included age, sex, educational level, physical activity level, lifetime history of depression, stroke, any cancer, myocardial infarction, hypertension, diabetes, congestive heart failure, BMI, smoking status, alcohol use, and cholesterol level. A knowledge-based approach was used to select the covariates for the full models from the information available. All-cause mortality was investigated as a possible competing risk event, but it was found not to be significantly associated with the eGFR after adjusting for relevant confounders; therefore, competing risk analyses were not included. The dose-response associations between kidney function measures and dementia (including subtypes) were assessed using restricted cubic spline functions with 4 knots at the 5th, 35th, 65th, and 95th percentiles of eGFRcr-cys.

Cross-sectional analyses were completed in a subsample of ESTHER participants with baseline p-tau181, GFAP, and NfL measurements. Linear regression analyses were completed with the plasma biomarkers as the dependent variables and kidney function (eGFRcr-cys) as the independent variable. The biomarkers were right skewed and therefore log transformed. Models with varying levels of adjustment as outlined were also used in these analyses. Correlation between the biomarkers and eGFRcr-cys was assessed with the Spearman correlation coefficients.

In addition, to investigate the possibility of reverse causation, logistic regression analyses were carried out with the log-transformed biomarker levels as the risk factors and cases of incident impaired kidney function as the outcome. Incident impaired kidney function was defined as participants with an eGFRcr-cys of 60 mL/min/1.73 m^2^ or more at baseline and an eGFRcr-cys of less than 60 mL/min/1.73 m^2^ at the 11-year follow-up.

Both the longitudinal Cox proportional hazards regression and cross-sectional linear regression analyses were also completed according to sex and age. All analyses were conducted using SAS software, version 9.4 (SAS Institute Inc). Statistical tests were 2-sided, and results were deemed statistically significant at *P* < .05.

## Results

### Participant Characteristics

This study included 6256 participants (3402 women [54.4%] and 2854 men [45.6%]; mean [SD] age at baseline, 61.7 [6.6] years [range, 50-75 years]]; 6255 participants [99.98%] were of European descent) (Table 1). A total of 510 participants received an all-cause dementia diagnosis within 17 years (cumulative incidence, 8.2%; incidence rate, 5.5 per 1000 person-years), while 5746 participants remained without a dementia diagnosis throughout follow-up. The mean (SD) length of follow-up was 10.9 (3.9) years for participants with incident dementia and 15.1 (3.4) years for participants who remained without a dementia diagnosis. The median age at dementia diagnosis was 78 years (IQR, 74-82 years). Kidney function was considered normal (≥90 mL/min/1.73 m^2^) in 2363 participants (37.8%), mildly decreased (60-89 mL/min/1.73 m^2^) in 3418 participants (54.6%), and impaired (<60 mL/min/1.73 m^2^) in 475 participants (7.6%). The unadjusted incidence of dementia differed by kidney function: 151 of 2363 participants with normal kidney function (cumulative incidence, 6.4%; incidence rate, 4.2 cases per 1000 person-years), 298 of 3418 participants with decreased kidney function (cumulative incidence, 8.7%; incidence rate, 6 cases per 1000 person-years), and 61 of 475 participants with impaired kidney function (cumulative incidence, 12.8%: incidence rate, 9.8 cases per 1000 person-years) (eFigure 2 in Supplement 1). The most common comorbidities were hypertension (3597 [57.5%]), diabetes (936 [15.0%]), and history of depression (919 [14.7%]) (Table 1). In addition, only one-fourth of participants had 10 years or more of formal education, and the mean (SD) BMI was 27.6 (4.3).

**Table 1.** Participant Characteristics at Baseline

Characteristic^a^	No. (%) (N = 6256)
Age, mean (SD) [range], y	61.7 (6.6) [50-75]
Sex	
Women	3402 (54.4)
Men	2854 (45.6)
Educational level, y	
≤9	4493 (73.5)
≥10	1618 (26.5)
eGFRcr-cys, mean (SD), mL/min/1.73 m^2^	84.2 (16.6)
eGFRcr-cys, mL/min/1.73 m^2^	
≥90	2363 (37.8)
60-89	3418 (54.6)
<60	475 (7.6)
BMI, mean (SD)	27.6 (4.3)
Physical activity level	
Inactive	1201 (19.2)
Medium	2832 (45.4)
Moderate or high	2208 (35.4)
Alcohol use	
Abstinent	1724 (36.7)
Light	3570 (54.7)
Moderate	326 (6.9)
Heavy	82 (1.7)
Smoking status	
Never	3126 (51.1)
Former	2063 (33.7)
Current	930 (15.2)
Lifetime history of depression	
Yes	919 (14.7)
No	5326 (85.3)
Stroke	
Yes	191 (3.1)
No	5881 (96.9)
Cancer	
Yes	375 (6.0)
No	5881 (94.0)
Myocardial infarction	
Yes	333 (5.5)
No	5754 (94.5)
Hypertension	
Yes	3597 (57.5)
No	2658 (42.5)
Diabetes	
Yes	936 (15.0)
No	5309 (85.0)
Congestive heart failure	
Yes	643 (10.3)
No	5581 (89.7)
NSAIDs	
Yes	918 (14.7)
No	5321 (85.3)
ACE inhibitors	
Yes	1037 (16.6)
No	5202 (83.4)
β-Blockers	
Yes	1355 (21.7)
No	4884 (78.3)
Calcium channel blockers	
Yes	811 (13.0)
No	5428 (87.0)
Diuretics	
Yes	1009 (16.2)
No	5230 (83.8)
Angiotensin receptor blockers	
Yes	340 (5.5)
No	5899 (94.5)
Statins	
Yes	527 (8.5)
No	5712 (91.5)
Cholesterol, mean (SD), mg/dL (n = 6255)	220.2 (51.1)
C-reactive protein, mean (SD), mg/dL (n = 6170)	0.4 (0.9)
8-iso-PGF_2α_ levels, mean (SD), nmol/mmol creatinine (n = 6137)	0.2 (0.3)
25(OH)D, mean (SD), ng/mL (n = 6097)	21.0 (9.4)
AD-related blood biomarker sample, mean (SD) (n = 766)	
Neurofilament light, pg/mL	18.2 (10.9)
Phosphorylated tau181, pg/mL	1.8 (1.3)
Glial fibrillary acidic protein, pg/mL	102.8 (63.2)

Abbreviations: ACE, angiotensin-converting enzyme; AD, Alzheimer disease; BMI, body mass index (calculated as weight in kilograms divided by height in meters squared); eGFRcr-cys, estimated glomerular filtration rate calculated with the use of the 2021 Chronic Kidney Disease Epidemiology Collaboration creatinine–cystatin C equation; NSAIDs, nonsteroidal anti-inflammatory drugs; PGF_2α_, prostaglandin F_2α_.

SI conversion factors: To convert total cholesterol to millimoles per liter, multiply by 0.0259; C-reactive protein to milligrams per liter, multiply by 10.0; and 25(OH)D to nanomoles per liter, multiply by 2.496.

^a^ No. (%) of missing values: educational level, 145 (2.3); BMI, 11 (0.2%); physical activity level, 15 (0.2%); alcohol use, 554 (8.9%); smoking status, 137 (2.2%); lifetime history of depression, 11 (0.2%); stroke, 184 (2.9%); myocardial infarction, 169 (2.7%); hypertension, 1 (0.03%); diabetes, 11 (0.2%); congestive heart failure, 32 (0.5%); all medications (NSAIDs, ACE inhibitors, diuretics, β-blockers, calcium channel blockers, angiotensin receptor blockers, and statins), 17 (0.3%); cholesterol level, 1 (0.03%); C-reactive protein level, 86 (1.4%); 8-iso-PGF_2α_ level, 119 (1.9%); 25(OH)D level, 159 (2.5%).

The dementia-related blood biomarker nested case-control sample included 766 participants: 261 participants with an incident dementia diagnosis and 505 participants who remained without a dementia diagnosis throughout 17 years of follow-up. The mean (SD) baseline level of NfL was 18.2 (10.9) pg/mL, the mean (SD) baseline level of p-tau181 was 1.8 (1.3) pg/mL, and the mean (SD) baseline level of GFAP was 102.8 (63.2) pg/mL (Table 1).

### Kidney Function and Dementia Risk

**Figure 1.**
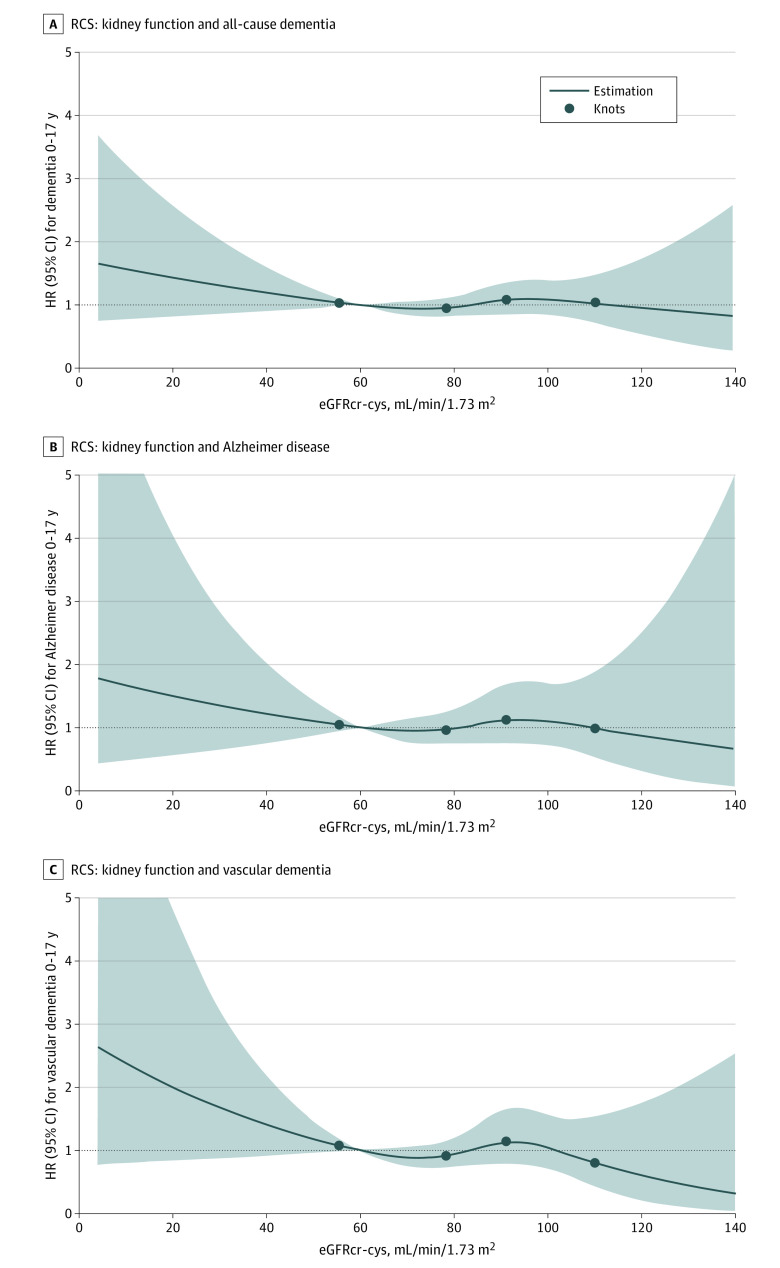
Dose-Response Association Between eGFRcr-cys and All-Cause Dementia, Alzheimer Disease, and Vascular Dementia Diagnosis Within 17 Years Adjusted for Age and Sex The study included 6256 total participants, 510 participants with incident dementia, 164 participants with incident Alzheimer disease, and 98 participants with incident vascular dementia. Restricted cubic spline (RCS) function of eGFRcr-cys levels are shown with 4 knots at the 5th, 35th, 65th, and 95th percentiles of biomarker levels and the median as the reference. eGFRcr-cys indicates estimated glomerular filtration rate calculated with the 2021 Chronic Kidney Disease Epidemiology Collaboration creatinine–cystatin C equation; HR, hazard ratio.

After extensive adjustment for relevant confounders, impaired kidney function at baseline (eGFRcr-cys <60 mL/min/1.73 m^2^ vs eGFRcr-cys ≥90 mL/min/1.73 m^2^) was not associated with a higher risk of all-cause dementia (HR, 0.77; 95% CI, 0.41-1.43), AD (HR, 0.98; 95% CI, 0.55-1.74), or vascular dementia diagnosis (HR, 1.05; 95% CI, 0.63-1.75) within 17 years (Table 2 and Figure 1). Similar associations were evident after adjustment for age and sex (all-cause dementia: HR, 0.95; 95% CI, 0.69-1.29; AD: HR, 0.94; 95% CI, 0.55-1.63; vascular dementia: HR, 1.06; 95% CI, 0.65-1.70). The unadjusted models did show significant associations between kidney function and dementia diagnosis risk; however, these associations were not present in models adjusted only for age (Table 2; eTable 2 in Supplement 1). There was no interaction between sex or age and kidney function in the association with dementia risk (eTable 3 and eTable 4 in Supplement 1). Finally, when restricting the sample to participants who also had available dementia-related blood biomarker measurements, similar results were evident (eTable 5 in Supplement 1).

**Table 2.** HRs for Incident Dementia Based on Baseline Kidney Function

Characteristic	Overall, No.	Cases, No.	Model 0^a^		Model 1^b^		Model 2^c^	
			HR (95% CI)	*P* value	HR (95% CI)	*P* value	HR (95% CI)	*P* value
**All-cause dementia**								
eGFRcr-cys, mL/min/1.73 m^2^								
≥90	2363	151	1 [Reference]	NA	1 [Reference]	NA	1 [Reference]	NA
60-89	3418	298	1.46 (1.20-1.78)^d^	<.001	0.83 (0.68-1.02)	.07	0.78 (0.57-1.08)	.14
<60	475	61	2.57 (1.91-3.46)^d^	<.001	0.95 (0.69-1.29)	.73	0.77 (0.41-1.43)	.41
**Alzheimer disease**								
eGFRcr-cys, mL/min/1.73 m^2^								
≥90	2363	49	1 [Reference]	NA	1 [Reference]	NA	1 [Reference]	NA
60-89	3418	95	1.44 (1.02-2.04)^d^	.04	0.82 (0.58-1.17)	.82	0.88 (0.61-1.26)	.48
<60	475	20	2.64 (1.57-4.44)^d^	<.001	0.94 (0.55-1.63)	.94	0.98 (0.55-1.74)	.93
**Vascular dementia**								
eGFRcr-cys, mL/min/1.73 m^2^								
≥90	2363	60	1 [Reference]	NA	1 [Reference]	NA	1 [Reference]	NA
60-89	3418	110	1.37 (1.00-1.88)	.05	0.77 (0.56-1.07)	.11	0.78 (0.56-1.09)	.15
<60	475	27	2.93 (1.86-4.62)	<.001^d^	1.06 (0.65-1.70)	.83	1.05 (0.63-1.75)	.84

Abbreviations: eGFRcr-cys, estimated glomerular filtration rate calculated by the 2021 Chronic Kidney Disease Epidemiology Collaboration creatinine–cystatin C equation; HR, hazard ratio; NA, not applicable.

^a^ Unadjusted.

^b^ Adjusted for age and sex.

^c^ Adjusted for age, sex, educational level, physical activity level, lifetime history of depression, stroke, any cancer, myocardial infarction, hypertension, diabetes, congestive heart failure, body mass index, smoking status, alcohol use, cholesterol level, C-reactive protein level, various medication use (nonsteroidal anti-inflammatory drugs, angiotensin-converting enzyme inhibitors, diuretics, β-blockers, calcium channel blockers, angiotensin receptor blockers, and statins), 8-iso-prostaglandin F_2α_ level, and 25(OH)D level.

^d^ Significant at *P* < .05.

### Kidney Function and Dementia-Related Blood Biomarkers

**Figure 2.**
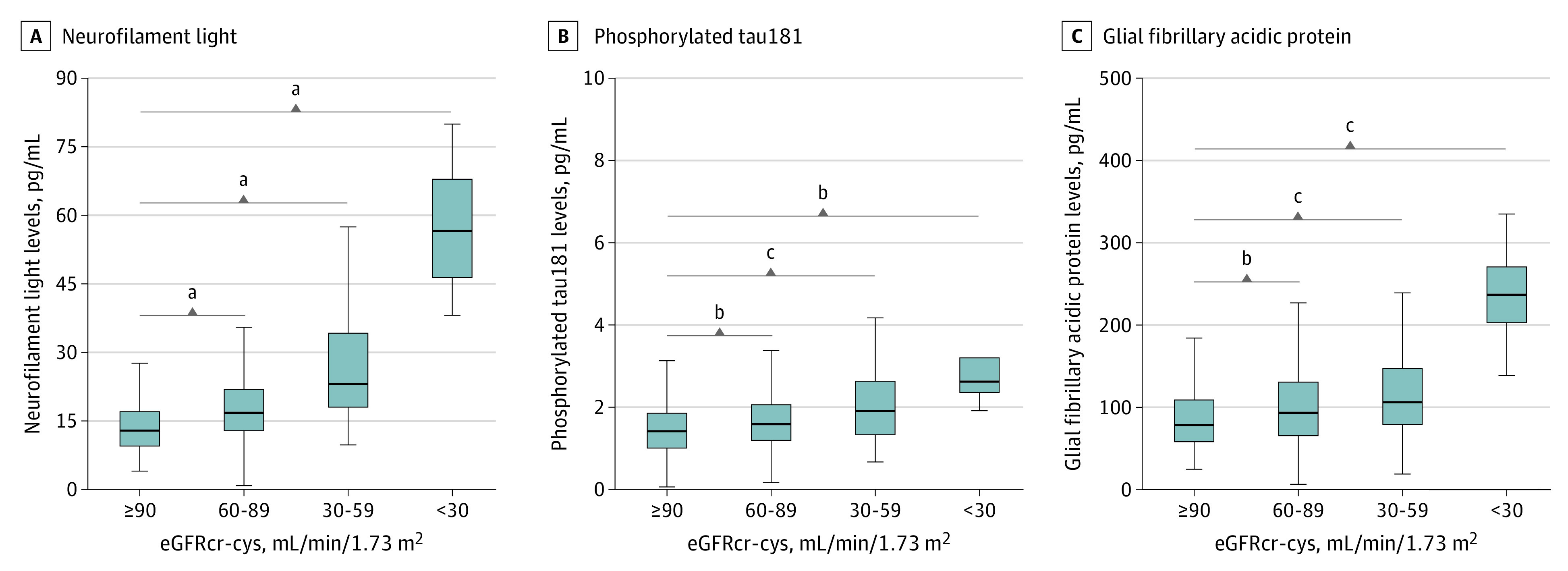
Dementia-Related Blood Biomarker Levels and Kidney Function Biomarker levels by kidney function (eGFRcr-cys, <30 mL/min/1.73 m^2^ [n = 4], 30-59 mL/min/1.73 m^2^ [n = 67], and 60-89 mL/min/1.73 m^2^ [n = 437] compared with ≥90 mL/min/1.73 m^2^ [n = 258]) are shown for neurofilament light, phosphorylated tau181, and glial fibrillary acidic protein. Mann-Whitney tests were used to compare for significant differences in biomarkers according to kidney function. eGFRcr-cys indicates estimated glomerular filtration rate calculated by the 2021 Chronic Kidney Disease Epidemiology Collaboration creatinine–cystatin C equation. The horizontal bar inside the boxes indicates the median, the lower and upper ends of the boxes indicate the lower and upper quartiles, and the whiskers indicate the minimum and maximum values excluding outliers. ^a^*P* < .001. ^b^*P* < .05. ^c^*P* < .01.

Mean dementia-related blood biomarker levels were significantly higher among participants with impaired kidney function compared with those with normal kidney function (Figure 2). All biomarkers exhibited significant correlations to each other as well as to eGFRcr-cys (NfL-GFAP: ρ = 0.49; NfL–p-tau181, ρ = 0.31; GFAP–p-tau, ρ = 0.33; eGFRcr-cys–NfL, ρ = −0.42; eGFRcr-cys–p-tau181, ρ = −0.22; eGFRcr-cys–GFAP, ρ = −0.21).

Impaired kidney function (eGFRcr-cys <60 mL/min/1.73 m^2^ vs eGFRcr-cys ≥90 mL/min/1.73 m^2^) was significantly associated with NfL and p-tau181 levels in blood after extensive adjustment for relevant confounders (NfL: β = 0.50 and *P* <. 001; p-tau181: β = 0.21 and *P* = .002) (Table 3). Similar associations were evident after adjusting for age and sex (NfL: β = 0.47 and *P* < .001; p-tau181: β = 0.21 and *P* = .003). The unadjusted models exhibited stronger associations between eGFRcr-cys and dementia-related blood biomarkers, with the association with GFAP also reaching significance, but were confounded by age as shown in the age-adjusted models (eTable 6 in Supplement 1).

**Table 3.** Association Between Blood Biomarkers and Kidney Function Including Sex-Specific Associations

Risk factor	No.	Model 0^a^		Model 1^b^		Model 2^c^	
		β Value	*P* value	β Value	*P* value	β Value	*P* value
**Outcome: log-transformed NfL**							
All							
eGFRcr-cys, mL/min/1.73 m^2^							
≥90	258	1 [Reference]	NA	1 [Reference]	NA	1 [Reference]	NA
60-89	437	0.27^d^	<.001	0.16^d^	<.001	0.17^d^	<.001
<60	71	0.70^d^	<.001	0.47^d^	<.001	0.50^d^	<.001
Men							
eGFRcr-cys, mL/min/1.73 m^2^							
≥90	123	1 [Reference]	NA	[1 Reference]	NA	1 [Reference]	NA
60-89	192	0.29^d^	<.001	0.19^d^	<.001	0.21^d^	<.001
<60	22	0.86^d^	<.001	0.71^d^	<.001	0.70^d^	<.001
Women							
eGFRcr-cys, mL/min/1.73 m^2^							
≥90	135	1 [Reference]	NA	1 [Reference]	NA	1 [Reference]	NA
60-89	245	0.27^d^	<.001	0.12^d^	.008	0.13^d^	.002
<60	49	0.65^d^	<.001	0.33^d^	<.001	0.35^d^	<.001
**Outcome: log-transformed p-tau181**							
All							
eGFRcr-cys, mL/min/1.73 m^2^							
≥90	258	1 [Reference]	NA	1 [Reference]	NA	1 [Reference]	NA
60-89	437	0.15^d^	.0001	0.09^d^	.02	0.11^d^	.01
<60	71	0.33^d^	<.001	0.21^d^	.003	0.21^d^	.002
Men							
eGFRcr-cys, mL/min/1.73 m^2^							
≥90	123	1 [Reference]	NA	1 [Reference]	NA	1 [Reference]	NA
60-89	192	0.16^d^	.006	0.12^d^	.03	0.14	.06
<60	22	0.27^d^	.02	0.21	.06	0.23	.05
Women							
eGFRcr-cys, mL/min/1.73 m^2^							
≥90	135	1 [Reference]	NA	1 [Reference]	NA	1 [Reference]	NA
60-89	245	0.15^d^	.008	0.06	.26	0.08	.16
<60	49	0.36^d^	<.001	0.13	.05	0.16	.09
**Outcome: log-transformed GFAP**							
All							
eGFRcr-cys, mL/min/1.73 m^2^							
≥90	258	1 [Reference]	NA	1 [Reference]	NA	1 [Reference]	NA
60-89	437	0.14^d^	<.001	−0.003	.94	−0.002	.95
<60	71	0.32^d^	<.001	0.02	.72	0.08	.20
Men							
eGFRcr-cys, mL/min/1.73 m^2^							
≥90	123	1 [Reference]	NA	1 [Reference]	NA	1 [Reference]	NA
60-89	192	0.16^d^	.01	0.04	.48	0.05	.38
<60	22	0.50^d^	<.001	0.31^d^	.006	0.42^d^	<.001
Women							
eGFRcr-cys, mL/min/1.73 m^2^							
≥90	135	1 [Reference]	NA	1 [Reference]	NA	1 [Reference]	NA
60-89	245	0.11	.04	−0.04	.37	−0.04	.39
<60	49	0.20^d^	.02	−0.12	.11	−0.12	.15

Abbreviations: eGFRcr-cys, estimated glomerular filtration rate calculated by the 2021 Chronic Kidney Disease Epidemiology Collaboration creatinine-cystatin C equation; GFAP, glial fibrillary acidic protein; NfL, neurofilament light; p-tau181, phosphorylated tau181; NA, not applicable.

^a^ Unadjusted.

^b^ Adjusted for age and sex (or age in the sex-stratified models).

^c^ Adjusted for age, sex, educational level, physical activity level, lifetime history of depression, stroke, any cancer, myocardial infarction, hypertension, diabetes, congestive heart failure, body mass index, smoking status, alcohol use, cholesterol level, C-reactive protein level, various medication use (nonsteroidal anti-inflammatory drugs, angiotensin-converting enzyme inhibitors, diuretics, β-blockers, calcium channel blockers, angiotensin receptor blockers, and statins), 8-iso-prostaglandin F_2α_ level, and 25(OH)D level.

^d^ Significant at *P* < .05.

The sex-stratified analyses revealed a sex-specific association between kidney function and GFAP with evidence of interaction (Table 3; eFigure 3 in Supplement 1). Impaired kidney function was only associated with GFAP levels among men in the fully adjusted model (men: β = 0.42; *P* < .001; women: β = −0.12 and *P* = .15). Similar associations were evident after adjusting for age and sex (men: β = 0.31 and *P* = .006; women: β = −0.12 and *P* = .11). Although all biomarkers were associated with age, only GFAP was also significantly associated with sex (eTable 7 in Supplement 1). In the age-stratified analyses, p-tau181 and GFAP were only significantly associated with eGFRcr-cys in the fully adjusted model among those aged 65 years or older (p-tau181: 50-64 years, β = 0.14 and *P* = .20; 65-75 years, β = 0.36 and *P* < .001; GFAP: 50-64 years, β = 0.04 and *P* = .71; 65-75 years, β = 0.27 and *P* = .007) (eTable 8 in Supplement 1).

In the reverse causation analyses, there were 22 cases of incident impaired kidney function at the 11-year follow-up (eTable 9 in Supplement 1). After adjusting for age and sex, none of the blood biomarkers were risk factors of impaired kidney function at the 11-year follow-up (NfL: odds ratio, 0.80; 95% CI, 0.48-1.33; p-tau181: odds ratio, 1.42; 95% CI, 0.94-2.14; GFAP: odds ratio, 0.88; 95% CI, 0.54-1.43).

## Discussion

Impaired kidney function, assessed by the eGFRcr-cys, was significantly associated with higher NfL and p-tau181 levels in blood, but not with risk of all-cause dementia, AD, or vascular dementia diagnosis in a community-based cohort prospectively followed up for 17 years. Sex-specific associations between kidney function and GFAP levels in blood samples were evident, with significant associations seen only among men.

### Kidney Function and Dementia Risk

Previous research findings regarding kidney function and dementia risk have been mixed. In several longitudinal studies, including the largest to date, an association between kidney function and increased risk of vascular dementia but not AD was evident.^4,22,23^ In a registry-based study, lower kidney function, measured by the eGFR, was associated with an increased risk of dementia as well as AD diagnosis.^3^ Longitudinal studies in Japan and England also showed associations between kidney function and incidence of dementia.^5,24^

However, many studies, including several large studies, did not illustrate an association between kidney function, measured by the eGFR, and risk of all subtypes of dementia.^6,7,8,25^ In a registry-based study^7^ as well as in follow-up of several longitudinal studies, a low eGFR was not associated with increased risk of any dementia.^6,8^ These results are in line with our study and support the lack of a definite association between kidney function and dementia, especially AD.

There are several theories linking poor kidney function to dementia, including the kidney-brain-axis theory that outlines the possible neuropathologic outcomes of poor kidney function.^26^ Increased dementia risk may be a result of the physiological consequences associated with constant blood flow in both the kidneys and the brain.^26^ Shared vascular risk factors may be important confounders in the association between kidney function and cognitive health, which could explain the uncertainty and mixed results regarding the association.

### Kidney Function and Dementia-Related Blood Biomarkers

Although, to our knowledge, limited research regarding kidney function and dementia-related blood biomarkers exists, a clear association between impaired kidney function and higher NfL levels in blood has been reported in the literature,^10,11,12^ as in our results. Chronic kidney disease,^10^ lower eGFR,^11^ and higher creatinine levels^12^ were associated with higher levels of NfL in blood. Previously, total tau, p-tau181, and p-tau217 levels have been associated with chronic kidney disease and creatinine levels.^10,12,13^ We present, for the first time, to our knowledge, an association between p-tau181 and NfL levels in blood and impaired kidney function as defined by the eGFR-cr-cys.

The association between blood GFAP levels and kidney function has not been previously investigated, to our knowledge. In our study, we found a sex-specific association, in which GFAP levels were associated with eGFRcr-cys only among men. Glial fibrillary acidic protein levels and kidney function have been shown to vary according to sex.^27,28^ Several studies have reported higher levels of GFAP among women,^28,29^ which was also evident in our study. Astrocytic response and neuroinflammation may be associated with sex hormones,^30^ which could be associated with GFAP levels.^31^ Estrogen is thought to aid in kidney repair and regeneration.^32^ Further research is necessary to confirm our findings and explore plausible mechanisms behind this association. In the age-stratified analyses, associations of eGFR with p-tau181 and GFAP levels were evident only among older individuals, possibly owing to increased rates of kidney function decline and AD neuropathology at older ages.

Reduced kidney function was associated with higher levels of dementia-related blood biomarkers. Reduced kidney clearance may result in increased circulating levels of the biomarkers in the blood. In our study, participants with severely impaired kidney function had higher blood biomarker levels and robust associations were evident even after consideration of confounders. In addition, we examined the possibility of reverse causation and did not find any association between baseline biomarker levels and incident impaired kidney function.

### Clinical Implications

The association of kidney function with dementia-related biomarkers has implications in the clinical translation of dementia-related biomarkers as prognostic or diagnostic markers of AD. Patients with impaired kidney function may have elevated dementia-related blood biomarkers that are not indicative of the true AD neuropathology present. Kidney function should be considered when determining clinically useful ranges to avoid the overestimation of neuropathology. To consider the use of dementia-related biomarkers as diagnostic or prognostic markers, it is important to further examine the impact of kidney function to provide the most accurate interpretation. Furthermore, kidney function should be considered in future research to determine the most accurate prognostic value of dementia-related blood biomarkers. For example, in the ESTHER study, the association between NfL level and AD became stronger and statistically significant only after adjusting for eGFRcr-cys in addition to age and sex (eTable 10 in Supplement 1).^19^

### Strengths and Limitations

This study has several strengths. To our knowledge, it is the first study to examine the association of kidney function with dementia risk and dementia-related blood biomarkers in the same the cohort, the first to use the eGFRcr-cys equation, the first to consider reverse causation between the dementia-related blood biomarkers and kidney function, and the first to explore the association with GFAP levels in blood. An additional strength includes the robust nature of the community-based prospective ESTHER study with a very long duration of follow-up.

This study also has some limitations, including the possibility of dementia misdiagnosis, underdiagnosis, or delayed diagnosis. The incidence of dementia was comparatively low in our study, potentially owing to underdiagnosis or participants’ younger age at baseline (50-75 years). The dementia diagnoses in the ESTHER study were clinical diagnoses reported heterogeneously by numerous practitioners, which is the nature of community-based cohort studies that portray common practice in such a setting. The strength of the diagnoses in the ESTHER study has been supported by previous work, in which the *APOE* ε4–AD polygenic risk score distribution among dementia diagnoses closely mirror that in the established literature.^17^ In addition, the limited number of dementia-related biomarker measurements and relatively small number of participants with an eGFRcr-cys less than 30 mL/min/1.73 m^2^ may limit the interpretation of the results. Finally, generalizability is limited to individuals of European descent.

## Conclusions

Impaired kidney function was associated with higher NfL and p-tau181 levels in blood, but not with AD or all-cause dementia risk in a prospective community-based cohort followed up for 17 years. A sex-specific association between kidney function and GFAP level was apparent, with significant associations seen only among men. The accuracy of dementia-related blood biomarkers may depend in part on kidney function, which should be considered in clinical translation. These findings should be confirmed, and the interaction between kidney function and sex in the association with GFAP level, including possible explanatory biological mechanisms, should be further explored in future research.

## Notes

Supplement 1.
eAppendix 1.
**eFigure 1.** Flow Chart of ESTHER Participants Included in the Study
eAppendix 2.
**eTable 1.** Imputed Variables and Percentage Missing **eFigure 2.** Cumulative Incidence of All-Cause Dementia, AD, and VD Diagnosis Within 17 Years by Kidney Function **eTable 2.** Cox Regression Results: HRs for Incident Dementia Based on Baseline Kidney Function in Age-Adjusted, Sex-Adjusted, and Restricted Full Models **eTable 3.** Cox Regression Results: HRs for Incident Dementia Based on Baseline Kidney Function Stratified by Sex **eTable 4.** Cox Regression Results: HRs for Incident Dementia Based on Baseline Kidney Function Stratified by Age **eTable 5.** Cox Regression Results: HRs for Incident Dementia Based on Baseline Kidney Function in AD-Related Blood Biomarker Subsample **eTable 6.** Age-Adjusted, Sex-Adjusted, and Restricted Full Model Linear Regression Results: Association Between Kidney Function and AD-Related Blood Biomarkers **eFigure 3.** Regression Lines for Each Biomarker, NfL [A], P-tau181 [B], and GFAP [C], With Kidney Function (eGFRcr-cys) Stratified by Sex **eTable 7.** Linear Regression Results: Association of Age and Sex With AD-Related Blood Biomarkers **eTable 8.** Linear Regression Results: Association Between Blood Biomarkers and Kidney Function According to Age **eTable 9.** Logistic Regression Results: ORs for Incident Impaired Kidney Function (eGFRcr-cys ≥60 mL/min/1.73 m^2^ at Baseline and eGFRcr-cys <60 mL/min/1.73 m^2 ^at the 11-Year Follow-up) **eTable 10.** Cox Regression Results: HRs for Incident Dementia Based on AD Related Biomarkers With Kidney Function Adjustment
eReferences.

Supplement 2.
Data Sharing Statement
