# Supplementary material for: Association of Kidney Function With Development of Alzheimer Disease and Other Dementias and Dementia-Related Blood Biomarkers
Source: JAMA Netw Open. 2023 Jan 24;6(1):e2252387. doi: 10.1001/jamanetworkopen.2022.52387 (PMC10408272; doi:10.1001/jamanetworkopen.2022.52387)
Supplement: Supplement 1. — eAppendix 1. eFigure 1. Flow Chart of ESTHER Participants Included in the Study eAppendix 2. eTable 1. Imputed Variables and Percentage Missing eFigure 2. Cumulative Incidence of All-Cause Dementia, AD, and VD Diagnosis Within 17 Years by Kidney Function eTable 2. Cox Regression Results: HRs for Incident Dementia Based on Baseline Kidney Function in Age-Adjusted, Sex-Adjusted, and Restricted Full Models eTable 3. Cox Regression Results: HRs for Incident Dementia Based on Baseline Kidney Function Stratified by Sex eTable 4. Cox Regression Results: HRs for Incident Dementia Based on Baseline Kidney Function Stratified by Age eTable 5. Cox Regression Results: HRs for Incident Dementia Based on Baseline Kidney Function in AD-Related Blood Biomarker Subsample eTable 6. Age-Adjusted, Sex-Adjusted, and Restricted Full Model Linear Regression Results: Association Between Kidney Function and AD-Related Blood Biomarkers eFigure 3. Regression Lines for Each Biomarker, NfL [A], P-tau181 [B], and GFAP [C], With Kidney Function (eGFRcr-cys) Stratified by Sex eTable 7. Linear Regression Results: Association of Age and Sex With AD-Related Blood Biomarkers eTable 8. Linear Regression Results: Association Between Blood Biomarkers and Kidney Function According to Age eTable 9. Logistic Regression Results: ORs for Incident Impaired Kidney Function (eGFRcr-cys ≥60 mL/min/1.73 m2 at Baseline and eGFRcr-cys <60 mL/min/1.73 m2 at the 11-Year Follow-up) eTable 10. Cox Regression Results: HRs for Incident Dementia Based on AD Related Biomarkers With Kidney Function Adjustment eReferences. [file jamanetwopen-e2252387-s001.pdf]

Supplementary Online Content

Stocker H, Beyer L, Trares K, et al. Association of kidney function with development of Alzheimer disease and other dementias and dementia-related blood biomarkers. *JAMA Netw Open.* 2023;6(1):e2252387. doi:10.1001/jamanetworkopen.2022.52387

eAppendix 1.
eFigure 1. Flow Chart of ESTHER Participants Included in the Study
eAppendix 2.
eTable 1. Imputed Variables and Percentage Missing
eFigure 2. Cumulative Incidence of All-Cause Dementia, AD, and VD Diagnosis Within 17 Years by Kidney Function
eTable 2. Cox Regression Results: HRs for Incident Dementia Based on Baseline Kidney Function in Age-Adjusted, Sex-Adjusted, and Restricted Full Models
eTable 3. Cox Regression Results: HRs for Incident Dementia Based on Baseline Kidney Function Stratified by Sex
eTable 4. Cox Regression Results: HRs for Incident Dementia Based on Baseline Kidney Function Stratified by Age
eTable 5. Cox Regression Results: HRs for Incident Dementia Based on Baseline Kidney Function in AD-Related Blood Biomarker Subsample
eTable 6. Age-Adjusted, Sex-Adjusted, and Restricted Full Model Linear Regression Results: Association Between Kidney Function and AD-Related Blood Biomarkers
eFigure 3. Regression Lines for Each Biomarker, NfL [A], P-tau181 [B], and GFAP [C], With Kidney Function (eGFRcr-cys) Stratified by Sex
eTable 7. Linear Regression Results: Association of Age and Sex With AD-Related Blood Biomarkers
eTable 8. Linear Regression Results: Association Between Blood Biomarkers and Kidney Function According to Age
eTable 9. Logistic Regression Results: ORs for Incident Impaired Kidney Function (eGFRcr-cys ≥60 mL/min/1.73 m^2 at Baseline and eGFRcr-cys <60 mL/min/1.73 m^2 at the 11-Year Follow-up)
eTable 10. Cox Regression Results: HRs for Incident Dementia Based on AD Related Biomarkers With Kidney Function Adjustment
eReferences.

This supplementary material has been provided by the authors to give readers additional information about their work.

eAppendix 1.

ESTHER participants completed standardized health questionnaires, provided blood and urine samples, and GPs provided medical information. Comprehensive monitoring of major disease incidence and mortality was conducted through participant and GP follow-up 2, 5, 8, 11, 14, and 17 years after recruitment for all participants. Furthermore, data were linked to the Saarland Cancer Registry and death certificates were obtained from local health authorities.

In order to collect information regarding dementia diagnoses throughout follow-up, questionnaires were sent to the GPs of all participants during the 14- and 17-year follow-ups. Dementia diagnosis information was sought for all participants, including those that had died during follow-up as outlined in **eFigure 1**. GPs were asked to fill out questionnaires regarding the participants' dementia status (i.e. presence of dementia diagnosis, type of dementia, dementia diagnosis date, or confirmed lack of dementia diagnosis) and provide all available medical records from specialists such as neurologists or psychiatrists. All-cause dementia diagnoses include unspecified or unknown dementia. The current guidelines in Germany for AD diagnosis follow the National Institute on Aging and the Alzheimer's Association[1], ICD-10, or the International Working group (IWG)-2 criteria, for VD diagnosis the National Institute of Neurological Disorders and Stroke (NINDS)- Association Internationale pour la Recherche et l'Enseignement en Neurosciences (AIREN) criteria,[2] and all-cause dementia diagnoses are recommended if the dementia symptoms outlined by the ICD-10 are present for at least six months.[3]

The AD diagnoses were reported by the participants' own GPs and in many cases participants had been explicitly referred to neurologists according to GP reports. Due to the setting of the study in the community, many different practitioners had been involved who had employed a variety of exams (including cranial CT scans and CSF exams), which were conducted and reported to a varying degree which reflects practice in routine care. The P-tau181 measurements were completed in blood from baseline (2000-2002, 0-17 years before diagnosis) that had been frozen at -80° C until the time of measurement (2020). The AD diagnoses were made before P-tau181 was measured and GPs did not see any blood biomarker (P-tau181, NfL, GFAP) results.

© 2023 Stocker H et al. *JAMA Network Open.*

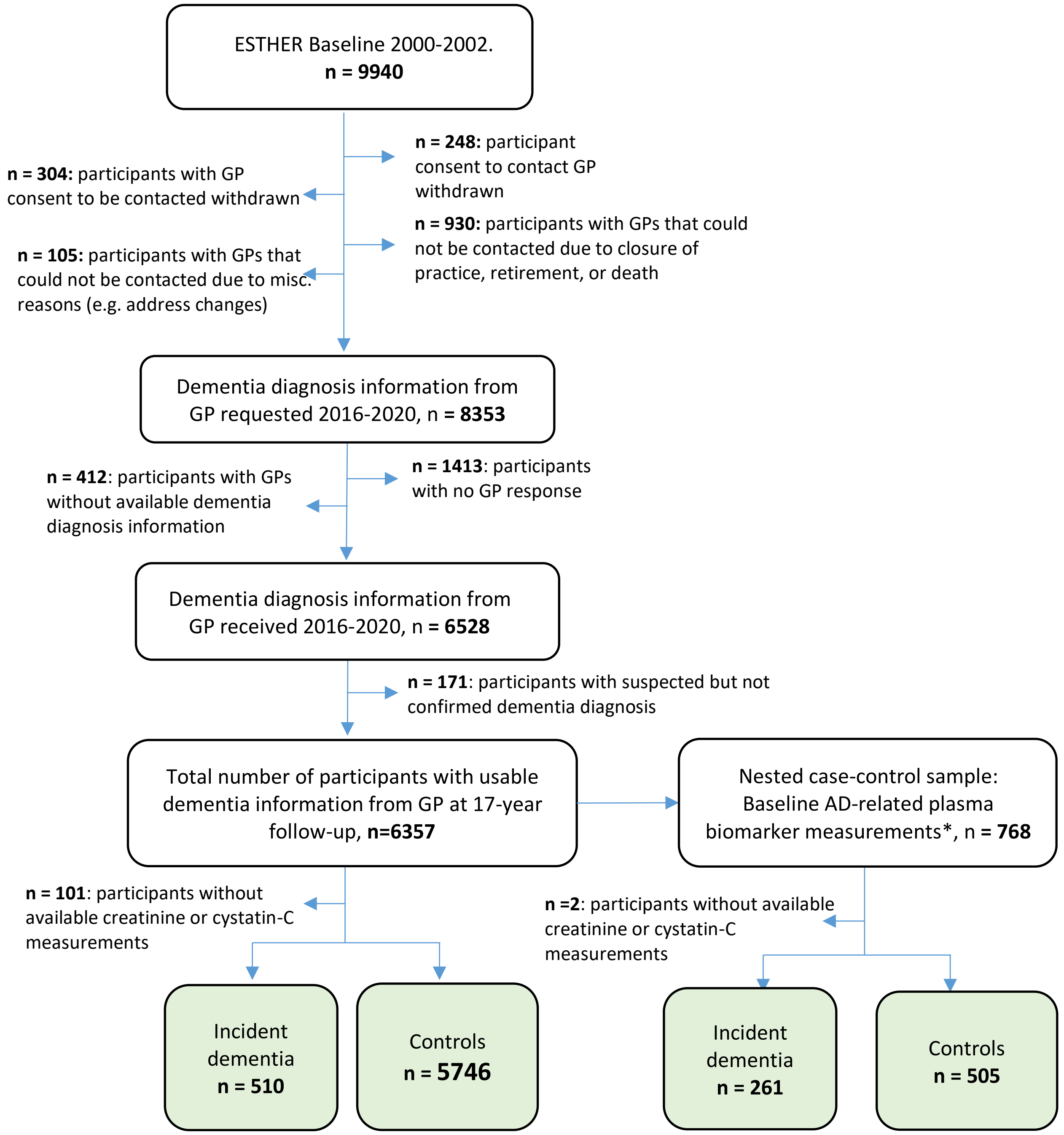

eFigure 1. Flow Chart of ESTHER Participants Included in the Study. Incident dementia cases included participants that received a dementia diagnosis between baseline and the 17-year follow-up. Controls included participants that remained without dementia diagnosis (GP confirmed) throughout follow-up. *P-tau181, GFAP, and NfL levels in blood plasma

© 2023 Stocker H et al. *JAMA Network Open.*

eAppendix 2.

Data ascertainment at baseline

The following information was ascertained at baseline through self-administered questionnaire and/or physician reports: age, sex, educational level, physical activity level, lifetime history of depression, stroke, any cancer, myocardial infarction, hypertension, congestive heart failure, body mass index (BMI), smoking status, alcohol use, and various medication use (NSAIDS (Anatomical Therapeutic Chemical Classification Codes B01AC06, N02BA01, N02BA51, N02BA71, M01A, M01BA01, M01BA03), ACE inhibitors (C09A, c09B), diuretics (C03), calcium-channel blockers (C08), beta blockers (C07, angiotensin receptors (C09C, C09D), and statins (C10AA)). Prevalent diabetes was defined as physician diagnosis, use of glucose lowering drugs or HbA1c ≥6.5% and fasting glucose ≥126 mg/dL or non-fasting glucose ≥ 200 mg/dL. Physical activity was defined as inactive: < 1 hour of physical activity/week, low: ≥ 1 hour of physical activity/week but < 2 hours of vigorous and < 2 hours of light physical activity/week, medium/high: ≥ 2 hours of light and ≥2 hours of vigorous physical activity/week. Alcohol use was defined by grams of ethanol per day with categories: abstainer, 0-19.99 g/d for women or 0-39.99 g/d for men, 20-39.99 g/d for women or 40-59.99 g/d for men, and ≥40 g/d for women or ≥60 g/d for men.

Laboratory measurements

Blood samples and urine samples taken at baseline were stored at −80°C until time of measurement. Serum creatinine measurements were performed by the kinetic Jaffe method. Serum cystatin C concentrations were measured by immunonephelometry on a Behring Nephelometer II (Dade-Behring Diagnostic, Marburg, Germany). C-reactive protein (CRP) was measured by immunoturbidimetry with the wrCRP antibody (Bayer, Leverkusen, Germany) on the ADVIA 2400. Total cholesterol and triglycerides measurements were determined from serum samples by a high-performance liquid chromatography method calibrated with the Synchron LX multicalibrator system (Beckman Coulter, Galway, Ireland). 25-hydroxy vitamin D (25(OH)D) concentrations, a marker of vitamin D status in baseline serum samples was measured using the automated Diasorin–Liaison analyzer (Diasorin, Inc.) and the automated IDS-iSYS analyzer (Immunodiagnostic Systems, GmbH). 8-iso-prostaglandin $F_{2\alpha}$ molecule (8-

© 2023 Stocker H et al. *JAMA Network Open.*

iso-$PGF_{2\alpha}$) levels (biomarker for lipid oxidation) were measured using 8iso1 enzyme-linked immunosorbent assay (ELISA) kits (Detroit R&D, Detroit, MI, USA) in spot urine samples.

Simoa technology was used to measure P-tau181, GFAP, and NfL in a single batch in plasma drawn at baseline as previously described.[4] Briefly, lithium-Heparin samples were measured utilizing the commercially available Simoa Neurology 4-Plex E Advantage Kit and Simoa pTau-181 Advantage V2 Kit (Quanterix, MA, USA) on the Simoa HD-X Analyzer according to manufacturer´s instructions at the Ruhr-University Bochum, Bochum, Germany. All laboratory measurements were performed in a blinded fashion.

© 2023 Stocker H et al. *JAMA Network Open.*

**eTable 1**. Imputed Variables and Percentage Missing	
Imputed Variable	% missing
education	2.3
depression	0.2
physical activiy	0.2
stroke	2.9
smoking status	2.2
hypertension	0.02
alcohol use	8.9
body mass index	0.2
myocardial infarction	2.7
congestive heart failure	0.5
diabetes	0.2
cholesterol	0.02
statins	0.3
nsaids	0.3
ace inhibitors	0.3
beta-blockers	0.3
diuretics	0.3
calcium-channel blockers	0.3
ARBs	0.3
C-reactive protein	1.3
8-iso-PGF2α	1.9
vitamin D	2.5

© 2023 Stocker H et al. *JAMA Network Open.*

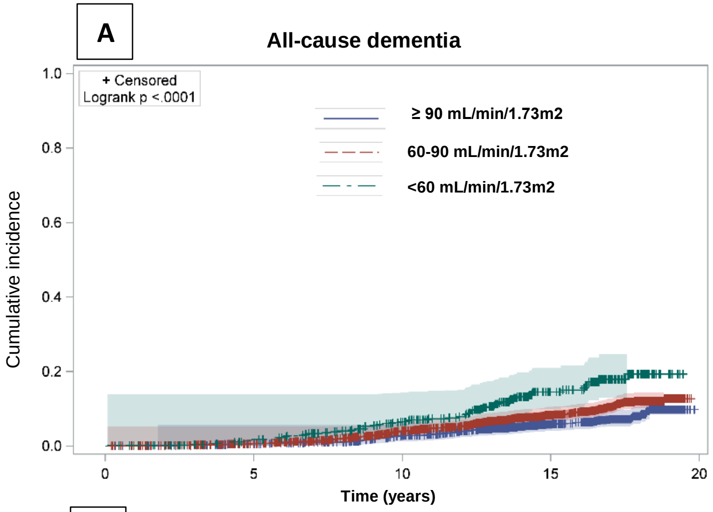

eFigure 2. Cumulative Incidence of All-Cause Dementia [A], AD [B], and VD [C] Diagnosis Within 17 Years by Kidney Function.

Kidney function was defined by eGFRcr-cys levels (≥ 90 mL/min/1.73m^2,60- 90 mL/min/1.73m^2, <60 mL/min/1.73m^2)

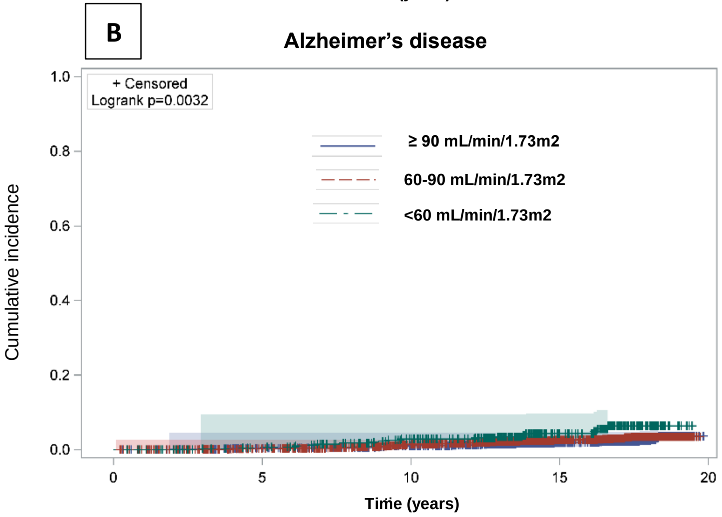

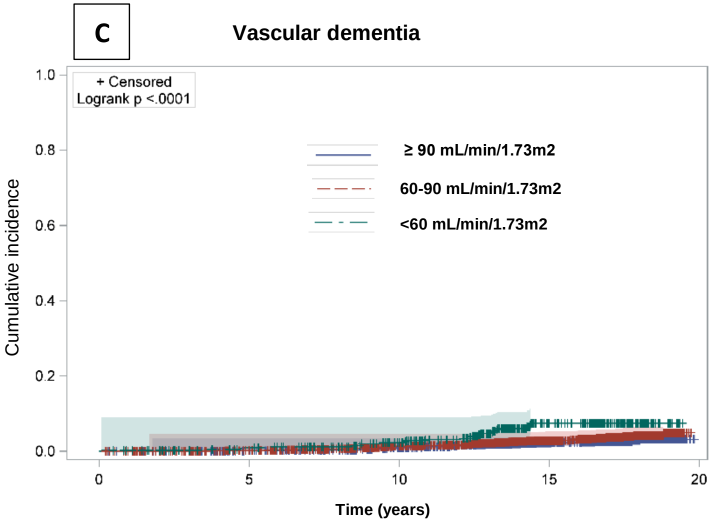

© 2023 Stocker H et al. *JAMA Network Open.*

eTable 2. Cox Regression Results: HRs for Incident Dementia Based on Baseline Kidney Function in Age-Adjusted, Sex-Adjusted, and Restricted Full Models

	Overall n	Cases n	Age adjusted HR (95% CI)	p-value	Sex adjusted HR (95% CI)	p-value	Full Model 1 HR (95% CI)	p-value
All-cause dementia								
eGFRcr-cys ≥ 90 mL/min/1.73m^2	2363	151	Ref.		Ref.		Ref.	
60-90 mL/min/1.73m^2	3418	298	0.83 (0.68-1.01)	.07	1.47 (1.21-1.79)	<.001	0.83 (0.68-1.02)	.08
< 60 mL/min/1.73m^2	475	61	0.93 (0.68-1.26)	.93	2.61 (1.93-3.51)	<.0001	0.90 (0.65-1.23)	.50
Alzheimer's disease								
eGFRcr-cys ≥ 90 mL/min/1.73m^2	2363	49	Ref.		Ref.		Ref.	
60-90 mL/min/1.73m^2	3418	95	0.83 (0.58-1.18)	.29	1.44 (1.02-2.03)	.04	0.85 (0.59-1.22)	.37
< 60 mL/min/1.73m^2	475	20	0.96 (0.56-1.65)	.87	2.62 (1.56-4.41)	<.001	0.94 (0.54-1.65)	.83
Vascular dementia								
eGFRcr-cys ≥ 90 mL/min/1.73m^2	2363	60	Ref.		Ref.		Ref.	
60-90 mL/min/1.73m^2	3418	110	0.77 (0.55-1.06)	.11	1.38 (1.01-1.89)	.045	0.74 (0.53-1.03)	.08
< 60 mL/min/1.73m^2	475	27	1.03 (0.64-1.66)	.90	2.98 (1.89-4.71)	<.0001	0.95 (0.58-1.55)	.82

Note: Full model 1 adjusted for age, sex, educational level, physical activity level, lifetime history of depression, stroke, any cancer, myocardial infarction, hypertension, diabetes, congestive heart failure, BMI, smoking status, alcohol use, and cholesterol level.
Bold values denote statistical significance at the p < .05 level.
Abbreviations: CI, confidence interval; eGFRcr-cys, estimated glomerular filtration rate according to the 2021CKD-EPI creatinine-cystatin C equation; HR, hazard ratio

© 2023 Stocker H et al. *JAMA Network Open.*

**eTable 3**. Cox Regression Results: HRs for Incident Dementia Based on Baseline Kidney Function Stratified by Sex								
	Overall n	Cases n	Model 0 HR (95% CI)		Model 1 HR (95% CI)		Model 2 HR (95% CI)	Interaction p-value
All-cause dementia								
Male								
eGFRcr-cys ≥ 90 mL/min/1.73m^2	1150	81	Ref.		Ref.		Ref.	.77
60- 90 mL/min/1.73m^2	1516	135	**1.35 (1.02-1.77)**		0.81 (0.61-1.07)		0.81 (0.59-1.11)	
< 60 mL/min/1.73m^2	188	25	**2.41 (1.54-3.78)**		1.08 (0.68-1.72)		1.00 (0.59-1.70)	
Female								
eGFRcr-cys ≥ 90 mL/min/1.73m^2	1213	70	Ref.		Ref.		Ref.	
60-90 mL/min/1.73m^2	1902	163	**1.61 (1.21-2.12)**		0.87 (0.65-1.15)		0.80 (0.58-1.11)	
< 60 mL/min/1.73m^2	287	36	**2.82 (1.89-4.22)**		0.86 (0.57-1.31)		0.63 (0.39-1.02)	
Alzheimer's disease								
Male								
eGFRcr-cys ≥ 90 mL/min/1.73m^2	1150	22	Ref.		Ref.		Ref.	
60- 90 mL/min/1.73m^2	1516	40	1.47 (0.87-2.47)		0.87 (0.51-1.50)		0.89 (0.50-1.56)	.84
< 60 mL/min/1.73m^2	188	6	2.18 (0.89-5.39)		0.94 (0.37-2.38)		0.90 (0.33-2.42)	
Female								
eGFRcr-cys ≥ 90 mL/min/1.73m^2	1213	27	Ref.		Ref.		Ref.	
60-90 mL/min/1.73m^2	1902	55	1.42 (0.89-2.24)		0.80 (0.50-1.29)		0.80 (0.48-1.31)	
< 60 mL/min/1.73m^2	287	14	**2.87 (1.50-5.48)**		0.96 (0.49-1.89)		0.77 (0.36-1.62)	
Vascular dementia								
Male								
eGFRcr-cys ≥ 90 mL/min/1.73m^2	1150	30	Ref.		Ref.		Ref.	.37
60- 90 mL/min/1.73m^2	1516	51	1.37 (0.87-2.15)		0.79 (0.49-1.26)		0.82 (0.50-1.34)	
< 60 mL/min/1.73m^2	188	13	**3.42 (1.79-6.57)**		1.44 (0.73-2.84)		1.29 (0.61-2.73)	
Female								
eGFRcr-cys ≥ 90 mL/min/1.73m^2	1213	30	Ref.		Ref.		Ref.	
60-90 mL/min/1.73m^2	1902	59	1.38 (0.89-2.14)		0.75 (0.48-1.18)		0.64 (0.39-1.03)	
< 60 mL/min/1.73m^2	287	14	**2.68 (1.42-5.05)**		0.84 (0.43-1.62)		0.54 (0.26-1.11)	

Note: Model 0 is unadjusted; Model 1 adjusted for age; Model 2 adjusted for age, educational level, physical activity level, lifetime history of depression, stroke, any cancer, myocardial infarction, hypertension, diabetes, congestive heart failure, BMI, smoking status, alcohol use, and cholesterol level, C-reactive protein, various medication use (NSAIDS, ACE inhibitors, diuretics, beta-blockers, calcium channel-blockers, ARBs and statins), 8-iso-PGF2α levels, and vitamin D (25(OH)D) levels.

© 2023 Stocker H et al. *JAMA Network Open.*

Bold values denote statistical significance at the $p < .05$ level.
Abbreviations: CI, confidence interval; eGFRcr-cys, estimated glomerular filtration rate according to the 2021CKD-EPI creatinine-cystatin C equation; HR, hazard ratio

© 2023 Stocker H et al. *JAMA Network Open.*

eTable 4. Cox Regression Results: HRs for Incident Dementia Based on Baseline Kidney Function Stratified by Age

	Overall n	Cases n	Model 0 HR (95% CI)		Model 1 HR (95% CI)		Model 2 HR (95% CI)	Interaction p-value
All-cause dementia								
50-64 years at baseline								
eGFRcr-cys ≥ 90 mL/min/1.73m^2	1867	77	Ref.		Ref.		Ref.	.19
60- 90 mL/min/1.73m^2	1949	68	0.86 (0.62-1.19)		0.87 (0.63-1.21)		0.81 (0.60-1.13)	
< 60 mL/min/1.73m^2	180	12	**1.89 (1.03-3.47)**		**1.92 (1.04-3.52)**		1.57 (0.84-2.94)	
65-75 years *at baseline*								
eGFRcr-cys ≥ 90 mL/min/1.73m^2	496	74	Ref.		Ref.		Ref.	
60-90 mL/min/1.73m^2	1469	230	1.12 (0.86-1.46)		1.26 (0.87-1.46)		1.15 (0.88-1.50)	
< 60 mL/min/1.73m^2	295	49	1.36 (0.95-1.96)		1.38 (0.96-1.98)		1.34 (0.92-1.95)	
Alzheimer's disease								
50-64 years at baseline								
eGFRcr-cys ≥ 90 mL/min/1.73m^2	1867	27	Ref.		Ref.		Ref.	
60- 90 mL/min/1.73m^2	1949	26	0.93 (0.54-1.60)		0.94 (0.55-1.60)		0.87 (0.50-1.52)	.69
< 60 mL/min/1.73m^2	180	2	0.89 (0.21-3.75)		0.89 (0.21-3.76)		0.78 (0.18-3.39)	
65-75 years *at baseline*								
eGFRcr-cys ≥ 90 mL/min/1.73m^2	496	22	Ref.		Ref.		Ref.	
60-90 mL/min/1.73m^2	1469	69	1.12 (0.70-1.82)		1.12 (0.69-1.81)		1.19 (0.73-1.93)	
< 60 mL/min/1.73m^2	295	18	1.65 (0.88-3.07)		1.61 (0.86-3.02)		1.75 (0.92-3.35)	
Vascular dementia								
50-64 years at baseline								
eGFRcr-cys ≥ 90 mL/min/1.73m^2	1867	30	Ref.		Ref.		Ref.	.31
60- 90 mL/min/1.73m^2	1949	24	0.78 (0.45-1.33)		0.78 (0.46-1.34)		0.68 (0.39-1.19)	
< 60 mL/min/1.73m^2	180	6	**2.45 (1.02-5.88)**		**2.47 (1.02-5.93)**		1.72 (0.69-4.32)	
65-75 years *at baseline*								
eGFRcr-cys ≥ 90 mL/min/1.73m^2	496	30	Ref.		Ref.		Ref.	
60-90 mL/min/1.73m^2	1469	86	1.03 (0.68-1.57)		1.04 (0.69-1.58)		1.04 (0.68-1.59)	
< 60 mL/min/1.73m^2	295	21	1.45 (0.83-2.53)		1.48 (0.85-2.59)		1.41 (0.79-2.52)	

Note: Model 0 is unadjusted, Model 1 adjusted for sex; Model 2 adjusted for sex, educational level, physical activity level, lifetime history of depression, stroke, any cancer, myocardial infarction, hypertension, diabetes, congestive heart failure, BMI, smoking status, alcohol use, and cholesterol level, C-reactive protein, various medication use (NSAIDS, ACE inhibitors, diuretics, beta-blockers, calcium channel-blockers, ARBs and statins), 8-iso-PGF2α levels, and vitamin D (25(OH)D) levels.
Bold values denote statistical significance at the p < .05 level.

© 2023 Stocker H et al. *JAMA Network Open.*

Abbreviations: CI, confidence interval; eGFRcr-cys, estimated glomerular filtration rate according to the 2021CKD-EPI creatinine-cystatin C equation; HR, hazard ratio

© 2023 Stocker H et al. *JAMA Network Open.*

**eTable 5**. Cox Regression Results: HRs for Incident Dementia Based on Baseline Kidney Function in AD-Related Blood Biomarker Subsample n=766								
	Overall n	Cases n	Model 0 HR (95% CI)	p-value	Model 1 HR (95% CI)	p-value	Model 2 HR (95% CI)	p-value
All-cause dementia								
eGFRcr-cys ≥ 90 mL/min/1.73m^2	258	71	Ref.		Ref.		Ref.	
60- 90 mL/min/1.73m^2	437	161	**1.45 (1.10-1.92)**	**<.01**	0.95 (0.72-1.26)	.72	0.93 (0.69-1.26)	.65
< 60 mL/min/1.73m^2	71	29	**1.89 (1.22-2.91)**	**<.01**	0.79 (0.50-1.24)	.30	0.77 (0.47-1.24)	.28
Alzheimer's disease								
eGFRcr ≥ 90 mL/min/1.73m^2	258	49	Ref.		Ref.		Ref.	
60- 90 mL/min/1.73m^2	437	95	1.29 (0.91-1.82)	.15	0.80 (0.55-1.15)	.22	0.89 (0.61-1.28)	.52
< 60 mL/min/1.73m^2	71	19	**1.88 (1.11-3.20)**	**.02**	0.69 (0.39-1.25)	.22	0.86 (0.48-1.56)	.62
Vascular dementia								
eGFRcr-cysy ≥ 90 mL/min/1.73m^2	258	20	Ref.		Ref.		Ref.	
60- 90 mL/min/1.73m^2	437	54	**1.80 (1.08-3.01)**	**.02**	1.19 (0.66-2.15)	.57	1.01 (0.58-1.73)	.98
< 60 mL/min/1.73m^2	71	9	2.15 (0.98-4.72)	.06	0.78 (0.32-1.92)	.58	0.76 (0.32-1.82)	.54
Note: Model 0 is unadjusted; Model 1 adjusted for age and sex; Model 2 adjusted for age, sex, educational level, physical activity level, lifetime history of depression, stroke, any cancer, myocardial infarction, hypertension, diabetes, congestive heart failure, BMI, smoking status, alcohol use, and cholesterol level, C-reactive protein, various medication use (NSAIDS, ACE inhibitors, diuretics, beta-blockers, calcium channel-blockers, ARBs and statins), 8-iso-PGF2α levels, and vitamin D (25(OH)D) levels. Abbreviations: CI, confidence interval; eGFRcr-cys, estimated glomerular filtration rate according to the 2021CKD-EPI creatinine-cystatin C equation; HR, hazard ratio								

© 2023 Stocker H et al. *JAMA Network Open.*

eTable 6. Age-Adjusted, Sex-Adjusted, and Restricted Full Model Linear Regression Results: Association Between Kidney Function and AD-Related Blood Biomarkers

Predictor	Age adjusted β	p-value	Sex adjusted β	p-value	Full model 1 β	p-value
Outcome: log-transformed NfL						
eGFRcr-cys ≥ 90 mL/min/1.73m^2	Ref.		Ref.		Ref.	
60- 90 mL/min/1.73m^2	**0.15**	**<.0001**	**0.28**	**<.0001**	**0.17**	**<.0001**
< 60 mL/min/1.73m^2	**0.46**	**<.0001**	**0.72**	**<.0001**	**0.50**	**<.0001**
Outcome: log-transformed P-tau181						
eGFRcr-cys ≥ 90 mL/min/1.73m^2	Ref.		Ref.		Ref.	
60- 90 mL/min/1.73m^2	**0.09**	**.02**	**0.15**	**<.0001**	**0.10**	**.01**
< 60 mL/min/1.73m^2	**0.22**	**<.01**	**0.33**	**<.0001**	**0.22**	**<.01**
Outcome: log-transformed GFAP						
eGFRcr-cys ≥ 90 mL/min/1.73m^2	Ref.		Ref.		Ref.	
60- 90 mL/min/1.73m^2	0.01	.89	**0.04**	**<.01**	0.005	.89
< 60 mL/min/1.73m^2	0.05	.40	**0.07**	**<.0001**	0.10	.13

Note: Full model 1 adjusted for age, sex, educational level, physical activity level, lifetime history of depression, stroke, any cancer, myocardial infarction, hypertension, diabetes, congestive heart failure, BMI, smoking status, alcohol use, and cholesterol level. Bold values denote statistical significance at the p < .05 level.
Abbreviations: eGFRcr-cys, estimated glomerular filtration rate according to the 2021CKD-EPI creatinine-cystatin C equation

© 2023 Stocker H et al. *JAMA Network Open.*

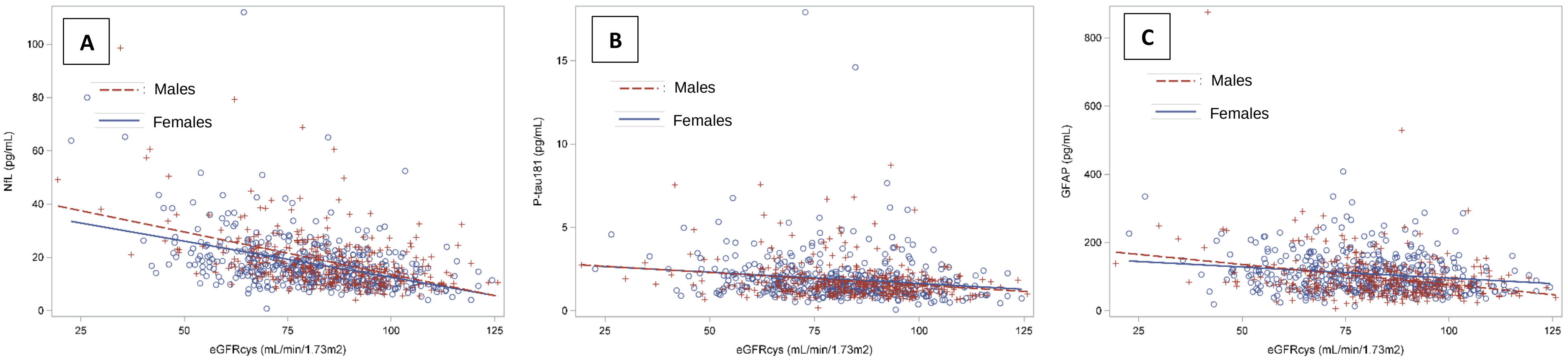

eFigure 3. Regression Lines for Each Biomarker, NfL [A], P-tau181 [B], and GFAP [C], With Kidney Function (eGFRcr-cys) Stratified by Sex

© 2023 Stocker H et al. *JAMA Network Open.*

**eTable 7.** Linear Regression Results: Association of Age and Sex With AD-Related Blood Biomarkers						
	log-transformed NfL		*log-transformed P-tau181*		*log-transformed GFAP*	
Predictor	β	p-value	β	p-value	β	p-value
Age	**0.04**	**<.0001**	**0.02**	**<.0001**	**0.04**	**<.0001**
Sex	0.05	.10	-0.04	.29	**-0.17**	**<.0001**
Note: Bold values denote statistical significance at the p < .05 level.						

© 2023 Stocker H et al. *JAMA Network Open.*

**eTable 8.** Linear Regression Results: Association Between Blood Biomarkers and Kidney Function According to Age							
Predictor	n	Model 0 β	p-value	Model 1 β	p-value	Model 2 β	p-value
Outcome: log-transformed NfL							
50-64 years							
eGFRcr-cys ≥ 90 mL/min/1.73m^2	181	Ref.		Ref.		Ref.	
60- 90 mL/min/1.73m^2	204	**0.18**	**<.0001**	**0.18**	**<.0001**	**0.20**	**<.0001**
< 60 mL/min/1.73m^2	21	**0.53**	**<.0001**	**0.53**	**<.0001**	**0.59**	**<.0001**
65-75 years							
eGFRcr-cys ≥ 90 mL/min/1.73m^2	77	Ref.		Ref.		Ref.	
60- 90 mL/min/1.73m^2	233	**0.18**	**.003**	**0.19**	**0.002**	**0.20**	**.0007**
< 60 mL/min/1.73m^2	50	**0.54**	**<.0001**	**0.56**	**<.0001**	**0.57**	**<.0001**
Outcome: log-transformed P-tau181							
50-64 years							
eGFRcr-cys ≥ 90 mL/min/1.73m^2	181	Ref.		Ref.		Ref.	
60- 90 mL/min/1.73m^2	204	**0.10**	**.03**	0.10	0.03	0.10	.04
< 60 mL/min/1.73m^2	21	0.13	.22	0.13	.21	0.14	.20
65-75 years							
eGFRcr-cys ≥ 90 mL/min/1.73m^2	77	Ref.		Ref.		Ref.	
60- 90 mL/min/1.73m^2	233	0.12	.09	0.11	.11	0.15	.04
< 60 mL/min/1.73m^2	50	**0.31**	**.001**	**0.29**	**.003**	**0.36**	**<.001**
Outcome: log-transformed GFAP							
50-64 years							
eGFRcr-cys ≥ 90 mL/min/1.73m^2	181	Ref.		Ref.		Ref.	
60- 90 mL/min/1.73m^2	204	0.05	.25	0.05	.27	0.06	.19
< 60 mL/min/1.73m^2	21	-0.009	.93	-0.02	.85	0.04	.71
65-75 years							
eGFRcr-cys ≥ 90 mL/min/1.73m^2	77	Ref.		Ref.		Ref.	
60- 90 mL/min/1.73m^2	233	0.04	.55	0.03	.67	0.05	.46
< 60 mL/min/1.73m^2	50	**0.23**	**.01**	**0.20**	**.04**	**0.27**	**<.01**
Note: Model 0 is unadjusted; Model 1 adjusted for sex; Model 2 adjusted for age, sex, educational level, physical activity level, lifetime history of depression, stroke, any cancer, myocardial infarction, hypertension, diabetes, congestive heart failure, BMI, smoking status, alcohol use, and cholesterol level, C-reactive							

© 2023 Stocker H et al. *JAMA Network Open.*

protein, various medication use (NSAIDS, ACE inhibitors, diuretics, beta-blockers, calcium channel-blockers, ARBs and statins), 8-iso-PGF2α levels, and vitamin D (25(OH)D) levels. Bold values denote statistical significance at the $p < .05$ level. Abbreviations: eGFRcr-cys, estimated glomerular filtration rate according to the 2021CKD-EPI creatinine-cystatin C equation

© 2023 Stocker H et al. *JAMA Network Open.*

**eTable 9**. Logistic Regression Results: ORs for Incident Impaired Kidney Function (eGFRcr-cys ≥60 mL/min/1.73m^2 at Baseline and eGFRcr-cys <60 mL/min/1.73m^2 at the 11-Year Follow-up)						
	Model 0<br>OR (95% CI)	p-value	Model 1<br>OR (95% CI)	p-value	Model 2<br>OR (95% CI)	p-value
n total=322, Incident impaired kidney function n=22						
NfL	1.32 (0.88-1.99)	.18	0.80 (0.48-1.33)	.39	0.69 (0.35-1.37)	.29
P-tau181	**1.69 (1.17-2.44)**	**<.01**	1.42 (0.94-2.14)	.09	1.35 (0.75-2.43)	.31
GFAP	1.30 (0.84-2.00)	.24	0.88 (0.54-1.43)	.60	1.07 (0.61-1.87)	.81

Note: All biomarkers are considered per standard deviation increase in log-transformed levels. Bold values denote statistical significance at the p < .05 level. Model 0 is unadjusted; Model 1 adjusted for age & sex; Model 2 adjusted for age, sex, educational level, physical activity level, lifetime history of depression, stroke, any cancer, myocardial infarction, hypertension, diabetes, congestive heart failure, BMI, smoking status, alcohol use, and cholesterol level, C-reactive protein, various medication use (NSAIDS, ACE inhibitors, diuretics, beta-blockers, calcium channel-blockers, ARBs and statins), 8-iso-PGF2α levels, and vitamin D (25(OH)D) levels.

Bold values denote statistical significance at the p < .05 level.

Abbreviations: CI, confidence interval; eGFRcr-cys, estimated glomerular filtration rate according to the 2021CKD-EPI creatinine-cystatin C equation; OR, odds ratio

© 2023 Stocker H et al. *JAMA Network Open.*

**eTable 10**. Cox Regression Results: HRs for Incident Dementia Based on AD Related Biomarkers With Kidney Function Adjustment				
	Age & sex adjusted HR (95% CI)	p-value	Age, sex & eGFRcr-cys adjusted HR (95% CI)	p-value
Outcome: Alzheimer's disease				
NfL	1.22 (0.98-1.51)	.07	**1.30 (1.03-1.63)**	**.03**
P-tau181	**1.19 (1.002-1.41)**	**<.05**	**1.22 (1.02-1.45)**	**.03**
GFAP	**1.58 (1.30-1.91)**	**<.0001**	**1.60 (1.31-1.94)**	**<.0001**
P-tau181				
Outcome: Vascular dementia				
NfL	**1.94 (1.48-2.55)**	**<.0001**	**1.98 (1.49-2.63)**	**<.0001**
P-tau181	1.18 (0.93-1.49)	.19	1.17 (0.92-1.49)	.19
GFAP	**1.36 (1.01-1.84)**	**.04**	**1.37 (1.01-1.85)**	**.04**

Note: All biomarkers are considered per standard deviation increase in log-transformed levels. Bold values denote statistical significance at the p < .05 level.
Abbreviations: CI, confidence interval; eGFRcr-cys, estimated glomerular filtration rate according to the 2021CKD-EPI creatinine-cystatin C equation; HR, hazard ratio

© 2023 Stocker H et al. *JAMA Network Open.*

eReferences.

1. McKhann GM, Knopman DS, Chertkow H, et al. The diagnosis of dementia due to Alzheimer’s disease: Recommendations from the National Institute on Aging-Alzheimer’s Association workgroups on diagnostic guidelines for Alzheimer's disease. *Alzheimers Dement*. 2011;7(3):263-269.
2. Román GC, Tatemichi TK, Erkinjuntti T, et al. Vascular dementia: diagnostic criteria for research studies: report of the NINDS-AIREN International Workshop. *Neurology*. 1993;43(2):250-250.
3. *S3-Leitlinie"Demenzen"*. 1 ed. Interdisziplinäre S3-Praxisleitlinien. Springer Berlin, Heidelberg 2016.
4. Stocker H, Beyer L, Perna L, et al. Association of plasma biomarkers, p-tau181, glial fibrillary acidic protein, and neurofilament light, with intermediate and long-term clinical Alzheimer's disease risk: Results from a prospective cohort followed over 17 years. *Alzheimers Dement*. Mar 2 2022;doi:10.1002/alz.12614

© 2023 Stocker H et al. *JAMA Network Open.*
