# Supplementary material for: Association of Kidney Function With Development of Alzheimer Disease and Other Dementias and Dementia-Related Blood Biomarkers
Source: JAMA Netw Open. 2023 Jan 24;6(1):e2252387. doi: 10.1001/jamanetworkopen.2022.52387 (PMC10408272; doi:10.1001/jamanetworkopen.2022.52387)
Supplement: Supplement 2. — Data Sharing Statement [file jamanetwopen-e2252387-s002.pdf]

Data Sharing Statement

Stocker. Association of Kidney Function With Development of Alzheimer Disease and Other Dementias and Dementia-Related Blood Biomarkers. *JAMA Netw Open*. Published January 24, 2023. doi:10.1001/jamanetworkopen.2022.52387

Data
Data available: No

Additional Information
Explanation for why data not available: Individual patient data will not be made available due to local regulations.
